# Knowledge, attitude and practice of infant feeding in the first 6 months among HIV-positive mothers at the Queen Mamohato Memorial hospital clinics, Maseru, Lesotho

**DOI:** 10.4102/phcfm.v10i1.1438

**Published:** 2018-05-17

**Authors:** Stephen O. Olorunfemi, Lilian Dudley

**Affiliations:** 1 Division of Community Health, Faculty of Medicine and Health Sciences, Stellenbosch University, South Africa

## Abstract

**Background:**

The balance between the risks of transmission of human immunodeficiency virus (HIV) through breastfeeding and its life-saving benefits complicates decisions about infant feeding among HIV-positive mothers in the first 6 months.

**Objective:**

The aim of this study was to assess the knowledge, attitude and practice of infant feeding among HIV-positive mothers attending the prevention of mother-to-child transmission services in Maseru, Lesotho.

**Method and setting:**

This observational cross-sectional study was done by collecting data from HIV-positive mothers attending the filter clinics of Queen Mamohato Memorial hospital in Maseru, Lesotho. HIV-positive mothers with infants below the age of 6 months attending the clinics at the time of the study were interviewed using a standardised questionnaire. We described the sociodemographic profile of the mothers, the information and education received on prevention of mother-to-child transmission (PMTCT) infant feeding options, the mothers’ knowledge, attitudes and practices of infant feeding, and assessed risk factors for improved knowledge, attitudes and practices.

**Results:**

The majority (96%) of the 191 HIV-positive mothers who participated in the survey knew about the PMTCT programme and related breastfeeding services. Most of the participants chose to breastfeed (89%), while only 8% formula-fed their infants. Knowledge received during the PMTCT programme was significantly associated with the decision to exclusively breastfeed their infants. Earlier infant feeding counselling and education was associated with more exclusively breastfeeding as compared to late infant feeding counselling (*p* < 0.001).

**Conclusion:**

The study found that HIV-positive mothers attending health clinics in Maseru, Lesotho, had high knowledge, and appropriate attitudes and practices with respect to infant feeding; and that early counselling and education improved infant feeding methods among these mothers.

## Introduction

The Joint United Nations Programme on HIV/AIDS (UNAIDS) reported in 2015 that there were an estimated 36.7 million people living with HIV globally, 1.8 million of whom were children. Of this population, 2.1 million were newly infected and 1.1 million died from the disease.^1^ Approximately 380 000 of the 1.89 million population of Lesotho are infected with HIV, of whom 38 000 were children between the age of 0–15 years and 342 000 were adults above the age of 15 years in 2014.^2^ Of an estimated 55 000 annual births in the country, approximately 15 235 infants were born to HIV-infected mothers in 2014.^2^

The prevention of mother-to-child transmission (PMTCT) of HIV programme was officially launched in Lesotho in February 2003, and it serves as an entry point for the prevention of HIV infection and care of HIV-infected women and their exposed infants. Currently there are 186 PMTCT sites, reflecting an increase in programme coverage from 16% in 2006 to 71% in 2009. This was made possible by the expanded training of health care providers, adoption of the provider-initiated testing approach, involvement of partners at implementation sites and the decentralisation of PMTCT services to health centre level in the entire country. PMTCT services are integrated into antenatal clinics (ANC) and all other maternal and child health (MCH) services.^3^ Health care providers educate these mothers on the importance of exclusively breastfeeding in addition to providing antiretroviral treatment (ART), which is referred to as Option B plus.^3^ Because of the transmissibility of HIV from mother to child during breastfeeding, the prevention of HIV infection during this period depends on appropriate feeding practices. This period has proven to be crucial in controlling the spread of the virus from HIV-positive mothers to their infants.^3^

In Lesotho, the general population of mothers who exclusively breastfed their infants under 6 months has increased over the last decade, from 36% in 2004 to 67% in 2014.^4^ Exclusive breastfeeding (EBF) declines with infant age: 44% of infants aged 4–5 months compared with 82% of infants aged 0–1 month and 76% of infants aged 2–3 months.^4^ Little research has been conducted in Lesotho to determine the prevalence of exclusive formula feeding (EFF) or mixed feeding (MF) in the population. The only study carried out among exposed infants in rural Lesotho in 2008 revealed that 76.7% of HIV-positive infants were exclusively breastfed, while 23.3% were exclusively formula-fed. The study did not report on MF.^5^

In 1999, De Cock et al. estimated that between 5% and 20% of infants would become infected if breastfed beyond 18 months.^6^ A meta-analysis by the Breastfeeding and HIV International Transmission Study Group found that 42% of infant infections were attributed to breast milk.^7^ Kourtis et al. calculated that without any intervention, approximately 6% of HIV-exposed infants would become infected via breastfeeding if they were exclusively breastfed for 6 months and rapidly weaned; 11% would become infected if they were mixed fed for 6 months, then rapidly weaned; and 15% would become infected if they were breastfed for 2 years.^8^ In the absence of effective interventions to prevent transmission of HIV during pregnancy, delivery or breastfeeding among HIV-infected pregnant women, it is estimated that 35% of births will result in MTCT of HIV.^6^ It is estimated that 3% of all under-5 mortalities in low-income countries could be prevented through mothers who exclusively breastfeed their infants during the critical first 6 months of life.^7^ Exclusively breastfeeding therefore plays a critical role in the overall health of infants, and is a global health objective, given its importance in the reduction of morbidity and mortality among infants, particularly in low-income countries where safe water and sanitation are often absent.^6^ The predicament concerning feeding infants of HIV-positive mothers is how to balance the risk of HIV transmission through breastfeeding with the risk of death from other causes other than HIV such as pneumonia, diarrhoeal diseases and malnutrition among formula-fed infants.^9^

The Government of Lesotho has implemented several HIV prevention strategies, including educational campaigns, work-based HIV prevention initiatives, the targeting of high-risk groups and PMTCT.^10^ All HIV-infected mothers attending health facilities in Lesotho receive information to assist them decide how to feed their infants, and to select the infant feeding option (IFO) that is most suitable to their situation. HIV---infected mothers in the country are also provided with Option B plus that was introduced in April 2013.^11^ It recommends providing lifelong ART to all pregnant and breastfeeding women living with HIV regardless of cluster of differentiation four (CD4) count or World Health Organization (WHO) clinical stage. ART is maintained for life after delivery and after the completion of breastfeeding.^1^

The PMTCT programme in Lesotho has made some advances in the past few years by creating great awareness of MTCT around the country.^1^ This was done through innovative strategies such as integration of ART for HIV-positive pregnant and lactating women and their infants in accessing services at the MCH unit, and with the integrated outreach community services. The use of community health workers to provide HIV testing, counselling and adherence support has allowed for early diagnosis, treatment and monitoring in the country’s remotest areas.^1^ Although the proportion of postnatal transmissions attributable to breastfeeding is not known exactly, it is likely to steadily decrease as the availability of prenatal and peripartum antiretroviral (ARV) prophylaxis continues to increase.^10,11^ Nevertheless, the successful reduction of MTCT remains dependent on adherence to EBF or replacement feeding (RF) of the baby for the first 6 months of life.^12^ The lack of appropriate knowledge, attitudes and practices (KAP) of infant feeding by HIV-positive mothers may therefore lead to an increase in MTCT of the virus.^2^

The following WHO or United Nations Children’s Fund or UNAIDS definitions are used for describing feeding options: EBF is defined as giving only breast milk and prescribed medicine, but no water, other liquids or food to infants. Exclusive replacement feeding (ERF) is the process of feeding a child who is not receiving any breast milk with a diet that provides all the nutrients the infant needs. Exclusive formula feeding is defined as feeding an infant only with prepared formula instead of the breast milk. Mixed feeding is defined as giving other liquids or foods together with breast milk to infants under 6 months of age.^10^ Replacement feeding is the provision of suitable breast milk substitutes that provide the necessary nutrients instead of breast milk, and include commercial infant formula (IF) or home-modified animal milk.^10^

The 2014 Lesotho National ARV guidelines recommend EBF for the first 6 months of an infant’s life, lifelong ARVs for the mother and Nevirapin (NVP) from birth to 6 weeks of the infant’s life.^2^ However, it provides specific conditions under which RF (with commercial IF) can be given to an HIV-exposed infant if the mother is on Option B plus. These include access to safe water and sanitation, a reliable supply of sufficient IF milk which the mother is able to prepare cleanly and frequently enough; the ability to exclusively give IF milk for the first 6 months, with full family support; and the ability to access health care that offers comprehensive child health services.

The above recommendations align with international recommendations for RF summarised as acceptable, feasible, affordable, sustainable and safe (AFASS).^10^ When AFASS criteria cannot be met, especially in a resource-challenged country with a high burden of HIV like Lesotho, HIV-positive mothers are advised to EBF and avoid MF the infants in the first 6 months.^11,12^

No previous studies have been conducted on the KAP of infant feeding among HIV-positive mothers in Lesotho. A South African study among 815 HIV-positive mothers attending postnatal clinics (mothers’ mean age was 27.7 years) showed that 71% knew that HIV could be transmitted during pregnancy, 78.7% knew that it could be transmitted during delivery and 78% agreed that it could be transmitted by breastfeeding. Half (50.6%) were EFF, 35.6% EBF and 12.4% reported MF. The study reported that 94.4% of women had received counselling on IFOs, although 14.7% did not receive counselling on IFOs within 72 h of delivery. Of the participants, 93% received information on the dangers of MF.^13^

A study in Kenya to assess the KAP with regard to infant feeding in the context of HIV showed that most of the respondents (85.5%) knew about breastfeeding as a route of HIV transmission. Breastfeeding was the custom in the society, but exclusive breastfeeding was not practiced among HIV-positive mothers. Cow’s milk, the main breast milk substitute, was reported as being given to infants as early as 2 weeks and was the most popular (93.5%) IFO in the context of HIVand AIDS.^14^

In Uganda, a study on the knowledge of MTCT of HIV among women enrolled in the PMTCT programme showed that out of 198 women, 26% believed that transmission of the virus is inevitable.^15^ A similar study in Ghana among HIV-positive mothers with infants aged 0–12 months showed that 33 (83%) out of the 40 HIV-positive mothers interviewed had received counselling on MTCT of HIV and WHO recommended feeding options during either antenatal care or postnatal services. Thirty-six participants (90%) mentioned the IFOs to include EBF and ERF. The majority of participants correctly understood EBF and only 10% partially understood ERF.^16^

Most of these studies demonstrated the major predictors of IFOs ranging from maternal attributes, mode of delivery and disclosure of HIV status to a spouse.

However, the most important factor was the knowledge acquired during the PMTCT programme.^17^ In addition, the information, education and counselling (IEC) provided to HIV-positive mothers during this period should include information about the risks and benefits of various IFOs and guidance in selecting the most suitable options for them and their infants.

Community views concerning the dangers of HIV transmission through breastfeeding and the discrimination associated with not breastfeeding make it difficult for HIV-positive mothers to initiate and maintain optimal infant feeding practices.^18^ Safe infant feeding in the context of HIV requires communication between parents and the whole family, as well as thorough, intensive community education, counselling and support.^19^

The aim of this study was therefore to determine the extent to which HIV-positive mothers attending PMTCT services in Lesotho complied with recommended infant feeding practices, and to identify factors that influenced their KAP.^6^

Self-reported maternal recall has been found to be a valid and reliable method of describing breastfeeding practices in cross-sectional studies, particularly for recall periods of less than 6 months.^20^ We therefore conducted a cross-sectional study of KAP of infant feeding of HIV-positive mothers with infants less than 6 months old.

## Methods

### Study design and setting

A cross-sectional study design was used to describe the infant feeding KAP of HIV-positive mothers registered at three primary health care facilities (Mabote, Qoaling and Likosi clinics) which refer patients to the Queen Mamohato Memorial hospital located in Maseru the capital city of Lesotho. The three clinics serve an estimated population of approximately 89 000 in the city of Maseru, which has a total population of about 4 million.^18,21^

### Sampling and data collection

Systematic sampling was employed to select HIV-positive mothers 18 years and older, with infants less than 6 months old, who visited and registered at the clinics during February and March 2015. Data collection was conducted using face-to-face interviews to complete a standardised questionnaire (Appendix 1) with each mother at the health facility. Mothers or infants with acute, life-threatening illness or neonatal deaths before 6 months were excluded from the study.

The desired sample size of 191 was estimated using Fisher’s formula, with the assumption of 95% confidence level, a margin of error of 5% and a prevalence value of knowledge of infant feeding based on western Kenya of 85.5%.^21^ The total number of questionnaires for the study was increased to 210, with an addition of 10% (19) (191 + 19) to allow for incomplete or unreliable answers from the participants. Five participants provided incomplete or incorrect responses which were corrected immediately in the field, and 14 incomplete questionnaires were discarded.

### Data handling and analysis

Three hospital nurses who were trained as research assistants conducted the interviews in the native language of the participants using a structured questionnaire which was adapted from an earlier WHO AIDS Questionnaire.^22^ A pilot study was conducted to pre-test the questionnaire with 15 HIV-positive mothers at the Mabote clinic. Each completed questionnaire was checked manually by the research assistants, and subsequently quality checked and captured into an Excel spreadsheet by the principal investigator (PI) at the end of each day. Inconsistent values were checked against the original questionnaire and corrected as necessary by the PI. The data were analysed using Stata version 13^23^ with the assistance of a statistician.

The analysis described the sociodemographic characteristics of participants, exposure to IEC on PMTCT and IFOs, and KAP of PMTCT and infant feeding. The results were organised in frequency counts, tables and graphs. With regard to KAP about breastfeeding in PMTCT, the number of correct responses by participants was categorised as either ‘high’ or ‘low’ knowledge.

Bivariate analysis and Fisher’s exact test or Pearson’s chi-squared test were used to assess factors associated with the KAP of IFOs. A two-tailed probability level of *p* < 0.05 was chosen as the level of statistical significance in the analysis.

Fisher’s exact test or Pearson’s chi-squared test was used to compare results between categorical data. Some groups were combined because of small numbers as follows:

- Completely satisfied or very satisfied or somewhat satisfied = Satisfied
- Somewhat dissatisfied or very dissatisfied = Dissatisfied
- No and do not know = No.

### Ethical considerations

Ethical approval (Ref#: S15/09/213) was obtained from the Health Research Ethics Review Committee of the Faculty of Medicine and Health Sciences, University of Stellenbosch, South Africa. Informed consent was obtained from all participants who agreed to be interviewed.

## Results

### Demographic profile and factors associated with practising exclusively breastfeed among the participants

Data were obtained from 191 HIV-positive mothers who had infants between the ages of 2 weeks and 6 months. The demographic profile of the respondents, including age, marital status, education, employment status and parity, is summarised in Table 1.

**TABLE 1a:** Demographic characteristics HIV-positive mothers attending prevention of mother-to-child transmission clinics in Maseru, Lesotho, 2015.

Demographic variables *N* = 191	*n*	%
**Age (years)**		
18–25	125	66
26–35	52	27
> 36	14	7
**Marital status**		
Single	25	13
Married	152	80
Other†	14	7
**Religion**		
Christian	183	96
Muslim	0	0
Traditional	6	3
Other	2	1
**Education**		
Primary or less	53	28
Secondary	117	61
Higher	21	11
**Employment**		
Government	19	10
Private	115	60
Self-employed	29	15
Unemployed	21	11
Student	7	4
**Parity**		
Primigravida (1)	100	52
Low parity (2–3)	70	37
High parity (≥ 4)	21	11

*N*, number.

† , other groups includes combined number of divorced, separated and widowed.

**TABLE 1b:** Information, education and communication (IEC) on mother-to-child transmission among HIV-positive mothers attending prevention of mother-to-child transmission clinics in Maseru, Lesotho, 2015.

Information, education and communication on infant (*N* = 191) feeding options	*n*	%
**When IEC on IFO was provided**		
Never	5	3
During pregnancy or immediate postpartum	169	88
Weeks postpartum	11	57
Months postpartum	6	3
**Nature of IEC on IFO**		
To breastfeed only	25	13
To formula feed only	15	26
To give both breastfeeding and formula	0	0
Counselled on both exclusive breastfeeding and exclusive formula feeding and asked to make a choice	151	79
**Satisfaction with IEC**		
Completely satisfied	76	40
Very satisfied	96	50
Somewhat satisfied	15	8
Somewhat dissatisfied	2	1
Very dissatisfied	2	1
Completely dissatisfied	0	0
**Main source of IEC**		
Partner	4	2
Mother	63	32
Friends	2	1
Nurse	120	62
Community health worker	2	1
**IFO advised against**		
Exclusive breastfeeding	0	0
Infant formula feed	5	3
Mixed feeding	166	87
Cow’s milk	20	10

IEC, Information, education and communication; IFOs, infant feeding options.

The majority of respondents (189, 99%) had their infant tested for HIV, of whom 187 (98%) were HIV negative, while two (1%) of the infants had not received the test report during the period of the study. The viral load level was not included in the study. Of the 191 mothers, only two had a caesarean section done at the main hospital, while all others had a normal vaginal delivery. Most (134, 70%) of the mothers confirmed that their partner was aware of their HIV status. Of the 191 respondents, 189 (99%) were on ART, of whom 132 (69%) started therapy during pregnancy, 38 (20%) were on treatment before their pregnancy and 19 (10%) started therapy after delivery. Only two (1%) of the mothers were yet to start therapy. Other attributes of the mothers with respect to IEC like IFO on type of feeding to avoid are included in Table 1.

### Knowledge, attitude and practice of infant feeding option

The various factors associated with KAP of HIV-positive in EBF their infants in the first six 6 months are displayed in Table 2.

**TABLE 2:** Knowledge, attitudes and practices in relation to infant feeding options among HIV-positive mothers attending prevention of mother-to-child transmission clinics in Maseru, Lesotho, 2015.

Variable	*n*	%
**A: Knowledge of MTCT and IFOs**		
**Infant feeding options (IFOs)**		
Cow’s milk	19	10
Infant formula only	29	15
Breastfeeding only	152	79
**Methods of reducing HIV in breast milk**		
Stop breastfeeding as soon as feasible in the first few months	5	3
Avoid mixed feeding or give only breast milk or formula	174	91
Heat treating milk	50	26
Give antiretroviral to the mother and child	175	92
**IFO with highest HIV transmission**		
Exclusive breastfeeding	3	16
Mixed feeding (giving both breast milk and formula)	185	97
Do not know	3	16
**Mother aware of the benefit of EBF**		
Yes	171	90
No	20	10
**B: Attitudes towards MTCT and IFOs**		
**EBF sufficient for 6 months**		
Yes	100	52
No	91	48
**Feel discriminated against in community about IFO**		
Yes	185	97
No	2	1
Don’t know	4	2
**C: Infant feeding practices**		
**Duration of EBF (months)**		
Less than 1 month	0	0
2–3 months	10	5
4–5 months	167	87
Still breastfeeding	14	7
Others	0	0
**Challenges with EFF**		
Expensive cost of purchasing formula milk	16	8
Pressure from relatives to breastfeed	160	84
No challenges	15	8
**Method of feeding used while formula feeding**		
Feeding bottle	10	67
Cup and spoon	3	20
Both feeding bottle and cup and spoon	2	13

MTCT, mother-to-child transmission; IFO, Infant feeding option; EBF, exclusive breastfeeding; EFF, exclusive formula feeding.

The mothers’ knowledge of MTCT through infant feeding was good. Most of the mothers (152, 80%) were aware of the two main IFOs (breastfeeding and IF), while only 39 (20%) had knowledge of other feeding options. Ninety-four per cent of the mothers (179) were aware of the risk of transmitting HIV to their infants during breastfeeding.

More than half (100, 52%) of the mothers agreed that breast milk alone was sufficient for the infant in the first 6 months, while 91 (48%) felt that this was not enough for proper growth.

The mothers’ knowledge of methods of HIV transmission during breastfeeding was categorised as ‘high’ if the participant responded correctly to two or more of the questions, or ‘low’ if the participant responded incorrectly to two or more of the questions. Using this summarised score of mothers’ knowledge about various methods of HIV transmission in MTCT, 76 (40%) of the participants gave one correct response, 65 (34%) gave two correct responses, and 50 (26 %) gave three correct responses.

The majority (176, 92%) of the HIV-positive mothers reported that even if provided with a free supply of IF, they would still prefer to exclusively breastfeed their infant, but 15 mothers (8%) preferred to formula feed their infants.

Most mothers (150, 78%) reported that their partners preferred them to EBF, but 30 (16%) partners preferred the mother to formula feed, while 11 (6%) mothers were not sure of the partner’s preference. About half (90, 47%) of the mothers agreed that infant feeding practices were decided by family members, while the rest of the mothers embraced the advice of the health staff (67, 35%), self (32, 17%) and spouse (2, 1%), respectively. More than half of the mothers (152, 79%) felt that EBF was the best IFO, while the rest of the mothers preferred other IFO.

Most of the mothers were not adherent to their choice of feeding. The study revealed that more than three-quarters (150, 78%) introduced other feeds apart from breast milk at about 5–6 months, while 26 (14%) EBF for more than 6 months, and 15 (8%) introduced other feeds earlier.

Most of the mothers (156, 82%) did not experience any challenges with EBF, while 11 (6%) claimed to not have enough breast milk, 10 (5%) had either cracked or sore nipples or were ill and 4 (2%) experienced pressure from relatives or friends to give water, formula or solid food to their infants.

Mothers indicated that family pressure and concerns that breast milk would not be enough for their infant were the main reasons for using IF or MF before 6 months. Of the 15 mothers who EFF their infant, 14 (93%) did so because of the worry of HIV transmission through breastfeeding, and 1 (6%) was too ill with a breast problem. Of these formula feeding mothers, 10 (67%) used piped or tap water for their IF preparation, and 5 (33%) used water from a closed well. All mothers who agreed to formula feed indicated that the water was boiled before use. More than three-quarters (160, 96%) will prefer to EBF if provided with IF.

There was a statistically significant relationship between the mother’s age (*p* < 0.001) and knowledge of methods of reduction of MTCT of HIV in infant feeding, with younger mothers aged 18–25 years being more knowledgeable. This did not however translate into a difference in EBF practices, with age not being associated with EBF at 4–5 months (*p* = 0.06).

The association between perception of the sufficiency of EBF in the first 6 months and mother’s age (*p* < 0.08) and mother’s education (*p* = 0.29) was not significant. The association between mothers’ age and their desire to exclusively breastfeed their infants for the first 4–5 months was not statistically significant (*p* = 0.06).

Knowledge about various feeding options and the level of satisfaction with the level of information provided during the programme was not statistically significant at *p* = 0.68, but the knowledge acquired about the various IFs compared with the time this information was given during this programme was statistically significant ( *p* < 0.0001).

Mothers who received information, education and counselling on IFO early during pregnancy were more likely to exclusively breastfeed as compared to mothers who received IEC late in their pregnancy or postnatally (*p* = 0.01). Mothers’ satisfaction with the IEC provided during the PMTCT programme was positively associated with EBF of infants (*p* = 0.006).

## Discussion

This study investigated infant feeding KAP of HIV-positive mothers at health clinics referring to Queen Mamohato Memorial hospital in Maseru, Lesotho. The high response rate and profile of mothers who participated in the study is typical of HIV-positive women in developing countries where MTCT of HIV is a public health challenge.^11^

The majority of the mothers in the study were aware of the benefits of EBF and preferred to exclusively breastfeed their infants, in agreement with their mothers-in-law’s and family advice. This is aligned with the strong cultural value attached to breastfeeding in the society.^24^

Most of the HIV-positive mothers were mindful of the risk of transmitting HIV to their infants during pregnancy, and the risk of MTCT associated with MF. This is similar to findings in other studies in sub-Saharan Africa, such as north-west Ethiopia, where most (89.5%) of the respondents had a good knowledge about EBF.^25^

The basic ethical principle of informed choice requires that HIV-positive mothers are provided with adequate information about their IFOs in the framework of PMTCT of HIV.^6^ The Lesotho National PMTCT guideline recommends that all pregnant mothers be counselled on infant feeding in order to make choices. The majority of the mothers were satisfied with the level of counselling and education provided on IFOs at the health facilities.

Early provision of IEC during pregnancy was associated with higher rates of EBF among the mothers.

Many health workers at the facilities had received training in the PMTCT programme a year prior to the study.^6^ The training provided by the International Centre for AIDS Care and Treatment Programmes (ICAP) in collaboration with the Elizabeth Glaser Paediatric AIDS Foundation may have contributed to the IEC provided by health services.^6^

Most mothers were aware that they could transmit HIV to their infants through breastfeeding, but other modes of transmission of HIV were generally less well known. This compares well to findings from a study in Ogun State, Nigeria, which revealed that only 27% of respondents understood the transmission of HIV during infant feeding.^24^

However, it was observed that three-quarters of the HIV-infected mothers understood that they need to make a choice between EBF and EFF. This gap suggests that there is some room for improvement with respect to education and counselling on infant feeding.

This outcome suggests a need to intensify health education on EBF among the mothers, with more emphasis on the goal and objectives of safe infant feeding practices. There was also overwhelming evidence that not all the mothers felt that breast milk was sufficient for the first 6 months. This implies that the knowledge about the sufficiency of breast milk for the first 6 months among mothers in the context of HIV and AIDS is still limited.

The proportion of MF in this study was 10.5%, which is lower than that reported by a study conducted in Ghana (40%),^17^ but closer to that of the study conducted in northern Ethiopia 6.3%.^26^ This outcome might be because of mothers providing ‘socially acceptable’ responses to the research assistants who were nurses in some of the health facilities. Cross-sectional studies, although valid and reliable measures of initiation and duration of breastfeeding, have been shown to overestimate EBF.^12^ Mothers may therefore not have accurately recalled specific instances of providing water, or dates of introducing solids, and also the grandparents are left to care for these infants while the mothers are at work, unfortunately these grandparents are not included in the PMTCT programme. Alternative appropriate methods for detailed studies of the dietary intake of infants less than 6 months which include the use of previous-day or seven-day recall periods.^27^

In order to assess the role of society members, mothers were asked about who influenced their choice of IFO and the source of information. The most common source of information regarding IFOs adhered to by the mothers was from their mothers-in-law, followed closely by the nurses at the health facilities. It is worth noting that the preferences of IFOs by mothers are those of the relatives despite the fact that the main source of information about IFO was from the health facilities.

This reinforces the difficulty women with HIV face in adhering to infant feeding in the face of the often over-whelming pressures that they face from their families. It supports other studies which found that parents of HIV-positive mothers and other key family members are very important in MTCT prevention programmes.^28^

Few of the mothers who did not breastfeed did so because they were worried about transmitting the virus to their infants. This is similar to other studies in India, where the majority of the mothers chose not to breastfeed for similar reasons.^29^

The pressure from relatives and friends to give water or adult foods to the infants has been attributed as the other challenges preventing HIV-positive mothers from exclusively breastfeeding their infant in the first 6 months. Therefore, there is a need for a multidimensional behavioural change strategy involving mothers and other family members. The finding that most participants felt that HIV-positive mothers are still experiencing discrimination in the community further reinforces the need for broader IEC and behavioural change strategies.

### Limitations

Only HIV-positive mothers attending the Queen Mamohato three filter clinics located in three large districts in Maseru were included in the study, limiting the generalisability of the study to the rest of Lesotho or other countries.

The maternal self-reporting method used in this study is a valid and reliable method of measuring knowledge and attitudes, as well as initiation and duration of breastfeeding particularly for short-term recall. However, it may overestimate EBF and underestimate MF practices. The involvement of health workers as research assistants may also have contributed to participants providing more ‘socially acceptable’ responses, leading to an overestimation of the desirable IFOs.

Lastly, as not all infants were 6 months old at the time of the survey, infant feeding practices may not reflect the actual 6-month practice of the mother infant pairs.

### Recommendations

The finding that some of the participants do not receive the recommended counselling on infant feeding underscores the need for the PMTCT programme in Lesotho. Although the Lesotho policy is aligned with international recommendations, the government needs to improve upon the present level of education among mothers and the communities so that they can make informed choices with respect to proper IFOs.

Further research is needed to understand the factors that affect women’s infant feeding decision-making, particularly the role of partners and mothers-in-law.

## Conclusions

HIV-positive mothers attending the PMTCT programme in Lesotho generally are knowledgeable and show a positive disposition to EBF their infants, with a clear understanding of the benefits of EBF.

Concerns about the sufficiency of EBF in the first 6 months was noted to be the major factor for mothers practising other forms of infant feeding. Therefore, the education and counselling concerning postnatal HIV transmission and safe IFOs should be adequate and clear.

The support of mothers-in-law or other key family members is an important factor in determining HIV-positive mothers’ infant feeding choices. Strong efforts should therefore be made to involve key family members in PMTCT programmes.

In summary, this study found that some mothers do not understand the risks of not practising correct IFOs, and confirmed that breastfeeding is considered a cultural norm as has been reported in similar settings of sub-Saharan Africa.^30^ HIV-positive mothers in Lesotho and similar settings need to be supported by the health care system, family and community in initiating and practising EBF for their HIV-exposed infants. Failure by policymakers to address these issues adequately will continue to lead to a gap between well-intended policies and programmes, and actual practices of HIV-positive mothers in Maseru, Lesotho.

## Appendix 1: Questionnaire

This question is purely for the purpose of this research. All information given will be treated as confidential and does not require names or identities of the informants. Kindly indicate with ‘X’ the most appropriate response (s) to the questions below.

Thank You.

**Table:**

**A) SOCIODEMOGRAPHIC CHARACTERISTICS**	
	**Please X correct answer**
Serial Number:	

Age of Mother: Sex of baby….. Baby date of birth………………	
Place of birth:	
**Date of interview:**	
**1. Age (as last birthday) in years: Age (years)**	
(a) 18–20	
(b) 21–30	
(c) 31–40	
(d) 41–49	
**2. Marital status**:	
(a) Married	
(b) Single	
(c) Divorced	
(d) Separated	
(e) Widowed	
(f) Cohabit	
**3. How many babies have you given birth to? ( Parity)**	
(a) primipara (1)	
(b) Low parity (2–3)	
(c) High parity (≥ 4)	
**4. Religion:**	
(a) Christian	
(b) Muslim	
(c) Traditional	
(d) Other (specify)	
**5. Education-level completed:**	
(a) None	
(b) Primary school	
(c) Secondary school	
(d) Higher than secondary	
(e) Unknown	
**6. Employment status**	
(a) Government employment	
(b) Privately employed	
(c) Self-employment	
(d) Unemployed or full-time housewife	
(e) Student	
(f) Volunteer	
**7. Does your spouse know about your HIV status?**	
(a) Yes	
(b) No	
**8. Has your baby been tested for HIV?**	
(a) Yes	
(b) No	
(c) If no, why was your baby not tested…?	
**9. If yes what is your baby’s HIV status?**	
(a) Positive	
(b) Negative	
**10. What are the methods by which HIV can be transmitted from mother to a child?**	
(a) During pregnancy	
(b) During delivery	
(c) During breastfeeding	
(d) Cannot be transmitted from mother-to-child	
(e) Do not know	
**11. Did you ever take antiretroviral (ARV)?**	
a) Yes	
b) No	
**12. If yes, when did you start ARVs?**	
(a) Never did	
(b) Only once before delivery	
(c) Have been on antiretroviral before and after pregnancy	
(d) Started after delivery	
(e) Started during pregnancy and continue after	
**13. Did your child ever take antiretroviral**?	
a) Yes	
b) No	
**14. If yes, when was the ARVs started after delivery?**	
(a) Only once after delivery	
(b) Started days after delivery	
(c) Immediately after delivery and continued for weeks or months	
(d) Continue throughout breastfeeding	
(e) Never did	
**B) KNOWLEDGE OF INFANT FEEDING OPTIONS**	
**15. How satisfied were you with the amount of education and counselling on infant feeding that you received?**	
(a) Completely satisfied	
(b) Very satisfied	
(c) Somewhat satisfied	
(e) Somewhat dissatisfied	
(f) Very dissatisfied	
(g) Completely dissatisfied	
**16. When did you receive any information about infant feeding options during pregnancy in the antenatal clinics (ANC)?**	
(a) Never received any information	
(b) During pregnancy or immediately after delivery	
(c) Few weeks after delivery	
(d) Months after delivery	
**17. What are the various infant feeding options you know? *(more than one allowed)**	
(a) Cow’s milk	
(b) Infant formula only	
(c) Surrogate mother only	
(d) Breastfeeding only	
(e) Heat treating express breast milk	
(f) Other	
**18. What advice were you given in terms of infant feeding during your ANC by the nurses or the counsellor?**	
(a) To breastfeed only	
(b) To formula feed only	
(c) To give both breastfeeding and formula	
(d) Counselled on both exclusive breastfeeding and exclusive formula feeding and asked to make a choice	
(e) Other	
**19. How can you reduce the risk of HIV transmission through breastfeeding?**	
(a) Stop breastfeeding as soon as feasible in the first few months	
(b) Avoid mixed feeding or given only breast milk or formula	
(c) Heat treating milk	
(e) Give antiretroviral to the mother and child	
(f) Other (specify)	
**20. Which infant feeding option has the highest risk of HIV transmission?**	
(a) Exclusive breastfeeding	
(b) Infant formula feed	
(c) Mixed feeding (giving both breast milk and formula milk)	
(d) Cow’s milk	
(e) Do not know	
**21. Do you know the benefit of exclusive breasffeeding (EBF)?**	

**C) ATTITUDE ABOUT INFANT FEEDING OPTIONS**	
**22. Will you have breastfed even with free supply of infant formula?**	
(a) Yes	
(b) No	
(c) I do not know	
**23. Which feeding option would you most like to be able to use?**	
(a) Exclusive breastfeeding	
(b) Infant formula feed	
(c) Mixed feeding (giving both breast milk and formula milk)	
(d) Cow’s milk	
(e) Do not know	
**24. Which feeding option does your partner feel you should use?**	
(a) Exclusive breastfeeding	
(b) Infant formula feed	
(c) Mixed feeding (giving both breast milk and formula milk)	
(d) Cow’s milk	
(e) Do not know	
**25. Which feeding option do other family members feel is the best to use?**	
(a) Exclusive breastfeeding	
(b) Infant formula feed	
(c) Mixed feeding (giving both breast milk and formula milk)	
(d) Cow’s milk	
(e) Do not know	
**26. Have you been told not to use any infant feeding options by family and friends?**	
(a) Yes	
(b) No	
**27. Which options have you been told not to use?**	
(a) Exclusive breastfeeding	
(b) Infant formula feed	
(c) Mixed feeding (giving both breast milk and formula milk)	
(d) Cow’s milk	
**28. Who do you listen to most about infant feeding?**	
(a) Partner	
(b) Mother	
(c) Partners	
(e) Family member	
(f) Friends	
(e) Nurse	
(f) CHW	
**29. Do you think breastfeeding alone is enough in the first 6 months for proper growth?**	
(a) Yes	
(b) No	
(c) I do not know	
**D) INFANT FEEDING PRACTICES**	
**30. In the first 6 months what feeding options did you give to your child?**	
**(participant can select more than one )**	
(a) Breastfeeding only (exclusive breastfeeding)	
(b) Breastfeeding and water	
(c) Formula feeding only	
(d) Breastfeeding, formula feeding and water	
(e) Heat-treated express milk	
(f) Other	
**31. Who decided on the feeding option(s) that you chose for your baby?**	
(a) Spouse	
(b) Self	
(c) Family member	
(d) Doctor or nurses	
(e) Other	
**32. How long did you exclusively breastfeed (breast milk alone)?**	
(a) Less than 1 month	
(b) 2–3 months	
(c) 4–6 months	
(d) Still breastfeeding	
(e) Others	
**33. At what age did you introduce other feeds apart from breast milk?**	
(a) From birth	
(b) 1–2 months	
(c) 3–4 months	
(d) 5–6 months	
(e) No other feeds in first 6 months	
**34. If you gave breast milk and formula or water or other diets before 6 months give reason?**	
(a) Breast milk not enough	
(b) Pressure from family or friends	
(c) Cost of purchasing formula	
(d) Ignorance of infant feeding option/HIV status	
(e) Other	
**35. How long did you give both breast and formula milk?**	
(a) Few days	
(b) Less than 1 month – 2 months	
(c) 2–4 months	
(d) Above 4 months	
(e) Presently still given both	
**36. What do you feel are the main problems or challenges with exclusive formula feeding?**	
(a) Expensive cost of purchasing formula milk	
(b) No regular supply of formula milk	
(c) Pressure from relatives or friends to give water and/or adult food	
(d) Problem with working and feeding	
(e) No challenges	
**37. What do you feel are the main problems or challenges with exclusive breastfeeding?**	
(a) Breast milk not enough for child	
(b) Pressure from relative or friends to give water or formula or adult diet	
(c) Crack or sore nipple or ill mother	
(d) No challenges	
(e) Other	
**E) FORMULA-FED MOTHER**	
**38. State the main reason why you did not breastfeed?**	
(a) Mother too ill or breast problem	
(b) Worry about transmitting HIV	
(c) Doctor or health provider advice	
(d) Family advice	
(e) Difficulty breastfeeding the infant	
(f) Others	
**39. If using formula milk, in your living condition, what kind of water is used for the infant formula preparation?**	
(a) Piped or tap water	
(b) Spring flowing	
(c) Close well	
(d) River or pond	
**40. If using formula milk what method of feeding is used?**	
(a) Feeding bottle	
(b) Cup and spoon	
(c) Both feeding bottle and cup or spoon	
(d) Other	
**41. Does discrimination against HIV-positive mothers in the community has any effect on your choice of infant feeding options?**	
(a) Yes	
(b) No	

THANK YOU FOR YOUR TIME.
